# Prospective Evaluation of Effect of Metformin on Activation of AMP-activated Protein Kinase (AMPK) and Disease Control in a Sub-group Analysis of Patients with GI Malignancies

**DOI:** 10.33696/Signaling.1.008

**Published:** 2020

**Authors:** Amandeep Godara, Nauman S. Siddiqui, Hilal Hachem, Philip N. Tsichlis, Robert E. Martell, Muhammad Wasif Saif

**Affiliations:** 1 Tufts Cancer Center, Tufts Medical Center, Boston, MA, USA; 2 Northwell Health Cancer Institute, Lake Success, NY, USA

**Keywords:** Metformin, Chemotherapy, mTOR, AMP-activated protein kinase (AMPK), Biguanide, Anti-diabetic, Diabetes, Cancer

## Abstract

**Background:**

Observational studies have demonstrated association of metformin with reduced cancer incidence and mortality in multiple cancer types, including gastrointestinal (GI) malignancies. Anti-neoplastic effects of metformin are believed through many mechanisms including activation of AMP-activated protein kinase, which controls mammalian target of rapamycin (mTOR) growth regulatory pathway.

**Methods:**

In a pilot, delayed-start randomized study, non-diabetic patients with GI cancers were randomized to 2 arms, Stage 1: concurrent metformin (500mg twice daily) plus chemotherapy vs. chemotherapy alone followed by cross over to metformin plus chemotherapy arm in Stage 2, while adverse events (DLT) were assessed by CTCAE v.3.0. As a translational correlate, we used phosphorylation of AMPKα at Thr172 to measure AMPK activation by western blot technique in PBMCs isolated from patients before and after receiving M. These levels were correlated with radiological (RECIST 1.1) and tumor marker outcomes by descriptive analysis. In this study, we present the sub-group analysis of patients with GI cancers.

**Results:**

41 patients with GI cancers (colorectal: 22, pancreatic: 12, gastroesophageal: 4, biliary: 2, others: 1) were treated in this trial. Mean duration of metformin therapy was 85 days (range: 9–443). There was no significant difference in grade 3 or above DLT in metformin plus chemotherapy vs. chemotherapy arm (14% vs. 12% respectively). Gel band density analysis on 19 patients showed that 63% patients had increased phosphorylation of AMPKα after metformin (ratio of phospho-AMPKα after and before metformin > 1) with mean = 1.227 (± 0.134). RECIST 1.1 restaging showed disease control in 55% patients and 45% patients had decline in tumor markers. Of note, 60% of patients with disease control also showed increase in phosphorylation of AMKα.

**Conclusions:**

This group of patients treated with metformin prospectively demonstrates the impact of metformin on AMPKα phosphorylation, and correlates with clinical benefit in patients with GI cancers when metformin was added to systemic chemotherapy of varying types. We aim to perform a dose-escalation of metformin in our next study with additional metabolomics correlates.

## Introduction

Observational studies have demonstrated that metformin, an anti-diabetic drug is associated with reduced cancer incidence and mortality in multiple cancer types [1–4]. Anti-neoplastic effects of metformin are believed to occur through many mechanisms including inhibition of mammalian target of rapamycin (mTOR) pathway by AMP-activated protein kinase (AMPKα) activation. mTOR pathway is involved in cellular growth and proliferation and is hyperactive in many cancers, therefore its inhibition could result in antitumor activity [5,6]. We previously published a phase I study to evaluate the safety of addition of metformin to systemic chemotherapy in patients with solid tumors [7]. In the current study, we pooled the data on patients with gastrointestinal (GI) cancers who were treated on our study and report the effect of metformin on disease control as well as activation of AMP-activated protein kinase (AMPKα).

## Patients and Methods

We conducted a delayed start randomized phase I clinical trial to explore the safety of adding metformin to chemotherapy in non-diabetic patients aged between 18–79 years with different solid and hematologic cancers as previously published [7]. In summary, to determine the safety of adding metformin to chemotherapy, we compared the incidence of dose-limiting toxicities (DLTs) in subjects receiving chemotherapy alone vs. in combination with concurrent metformin. An initial run-In Stage was held to establish a well-tolerated chemotherapy dosing regimen and to diminish confounding variables in toxicity. In the following stage, Stage 1, we randomized each patient to one of two arms, either a concurrent arm (metformin with chemotherapy) versus a delayed metformin arm (chemotherapy alone for Stage 1). This allowed a direct comparison of safety in patients receiving either chemotherapy alone versus with metformin. In the final stage, Stage 2, both arms then received metformin concurrently with chemotherapy. Metformin was given at a dose of 500 mg twice daily. Finally, we conducted an initial safety analysis by comparing the incidence of DLTs, adverse events ≥ Grade 3 in participants in the concurrent arm versus the delayed metformin arm. Tumor markers (CA 19–9, CEA) were measured at visits for response evaluation.

As a translational correlate, phosphorylation of AMPKα at Thr172 was used as a marker of AMPK activation in peripheral blood mononuclear cells (PBMC) [7–9]. Blood was collected from patients before and after receiving metformin in heparinized vacutainer tubes and PBMC were isolated by density gradient centrifugation. Cells were washed with PBS and then freezed. Frozen cells were lysed with lysis buffer supplemented with protease and phosphatase inhibitors. Sodium dodecyl sulfate polyacrylamide gel electrophoresis (SDS-PAGE) was then performed and protein bands were transferred onto polyvinylidene difiuoride (PVDF) membranes. The membranes were probed with antibodies specific to phospho-AMPKα (Thr172), total AMPKα, and α-tubulin as a loading control. Quantification of the bands was done using Image J. Intensities of the phospho-AMPKα bands were adjusted to the intensities of the total AMPKα bands, and the intensities of the total AMPKα bands were adjusted to the intensities of the α-tubulin bands to generate adjusted density values. Subsequently, adjusted density values after metformin treatment were divided by the adjusted density values before the metformin treatment to generate the ratio of phospho-AMPKα or total AMPKα after metformin to before metformin. Ratio greater than 1 will indicate an increase in the phosphorylation of AMPKα or total level of AMPKα after metformin treatment, and ratio lesser than 1 will indicate a decrease in the AMPKα phosphorylation or AMPKα level after treatment with metformin. Then these levels were correlated to radiological outcomes (RECIST criteria) by using descriptive analyses [10].

## Results

For this analysis, we identified all the patients with histologically confirmed diagnosis of GI malignancies treated on phase I study. Forty-one patients with GI malignancies (colorectal: 22, pancreatic: 12, gastroesophageal: 4, biliary: 2, others: 1) were identified in this trial (Table 1). Mean duration of metformin therapy was 85 days (range, 9–443 days).

The chemotherapy regimens consisted of various agents, including:

- Platinum (cisplatin, carboplatin, oxaliplatin)
- Antimicrotubule agents (paclitaxel, docetaxel, nab-paclitaxel)
- Anthracyclines (doxorubicin, epirubicin)
- Antibodies (cetuximab, trastuzumab, panitumumab, bevacizumab)
- Topoisomerase agents (irinotecan, etoposide)
- Tyrosine kinase inhibitors (erlotinib, imatinib, lapatinib, sorafenib, sunitinib)
- Alkylating agents (temozolomide)
- Antimetabolites (gemcitabine, 5-fiuorouracil, capecitabine)

Adverse events ≥ Grade 3 had a higher incidence in the concurrent arm (15.7 vs. 13.6%) in Stage 1 but a lower incidence in Stage 2 (6.2% vs. 9.5%) No lactic acidosis, a known AE associated with metformin, occurred in any patient (Table 2 and 3).

**Figure 1:**
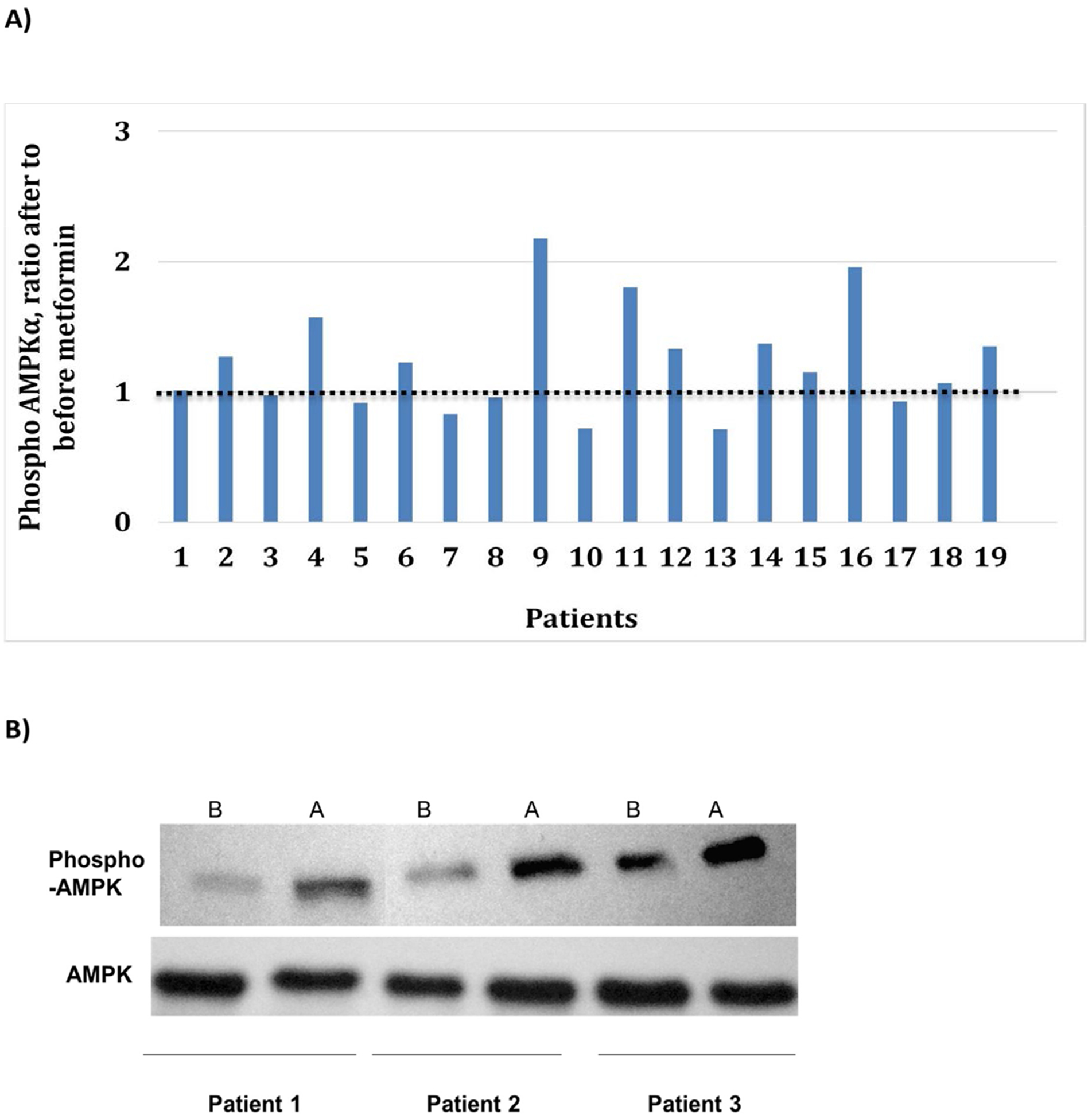
Effect of Metformin on AMPK phosphorylation in PBMCs. **A)** Evaluation of AMPKα in peripheral blood mononuclear cells (PBMC). Bar diagram shows the ratio of phospho-AMPKα after metformin to before metformin for all evaluable patients. Ratio greater than 1 (dashed line) indicates an increase in the phosphorylation of AMPKα or total level of AMPKα after metformin treatment, and ratio lesser than 1 indicates a decrease in the AMPKα phosphorylation or AMPKα level after treatment with metformin. **B)** Effect of Metformin on Phosphorylation of AMPKα in PBMCs from representative patients, before (B) and after (A) metformin.

Gel band density analysis on 19 random patients showed that 63% patients had increase in phosphorylation of AMPKα after metformin (ratio of phospho-AMPKα after metformin to before metformin >1) with the mean = 1.227 (± 0.134). Also, 60% of patients with stable disease had an increase in phosphorylation of AMKα (Figures 1A and 1B).

**Figure 2:**
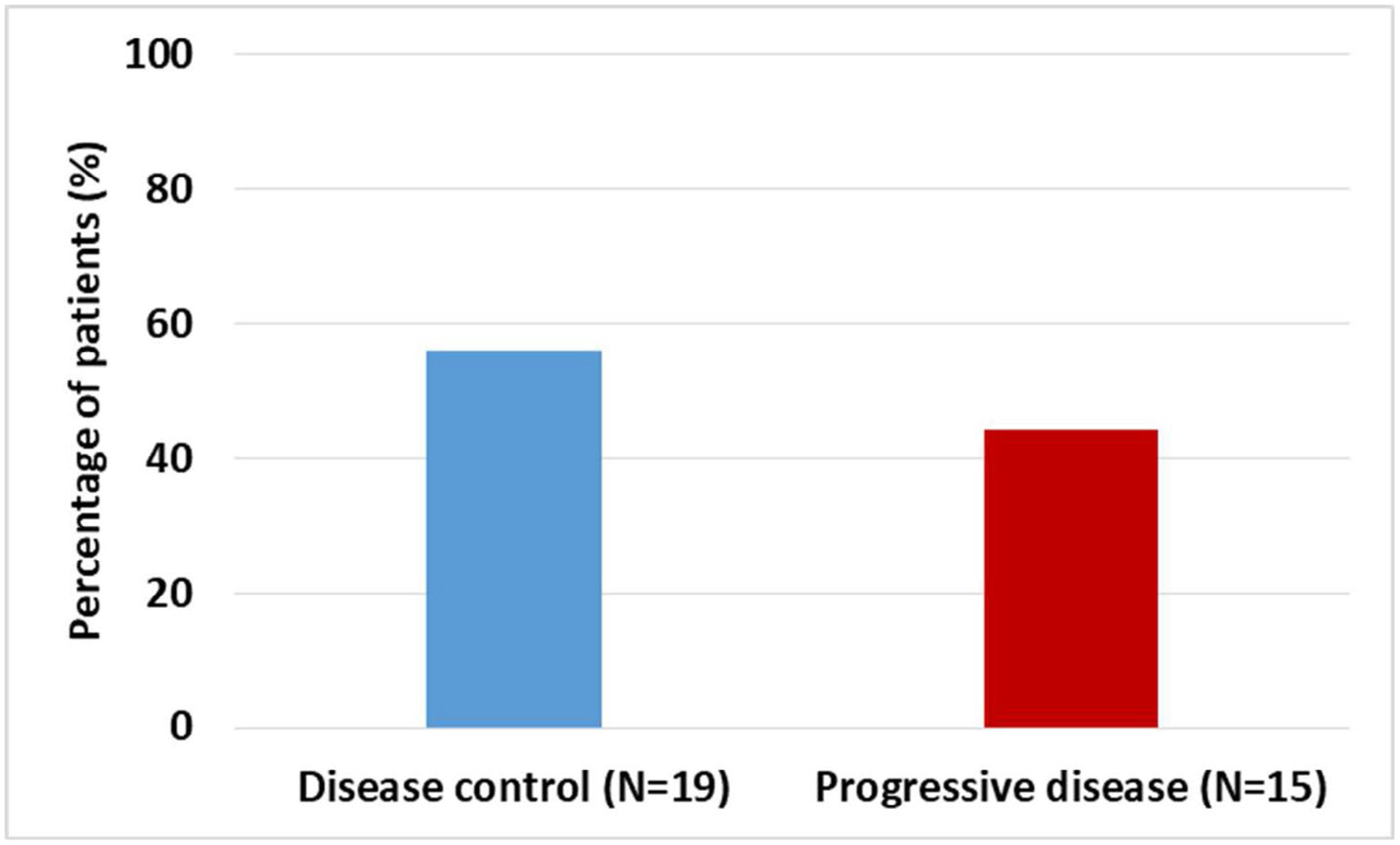
Disease response by radiology evaluation. Bar diagram showing outcomes of radiologic evaluation post-treatment. Fifty-five percent of patients had disease control or better response.

**Figure 3:**
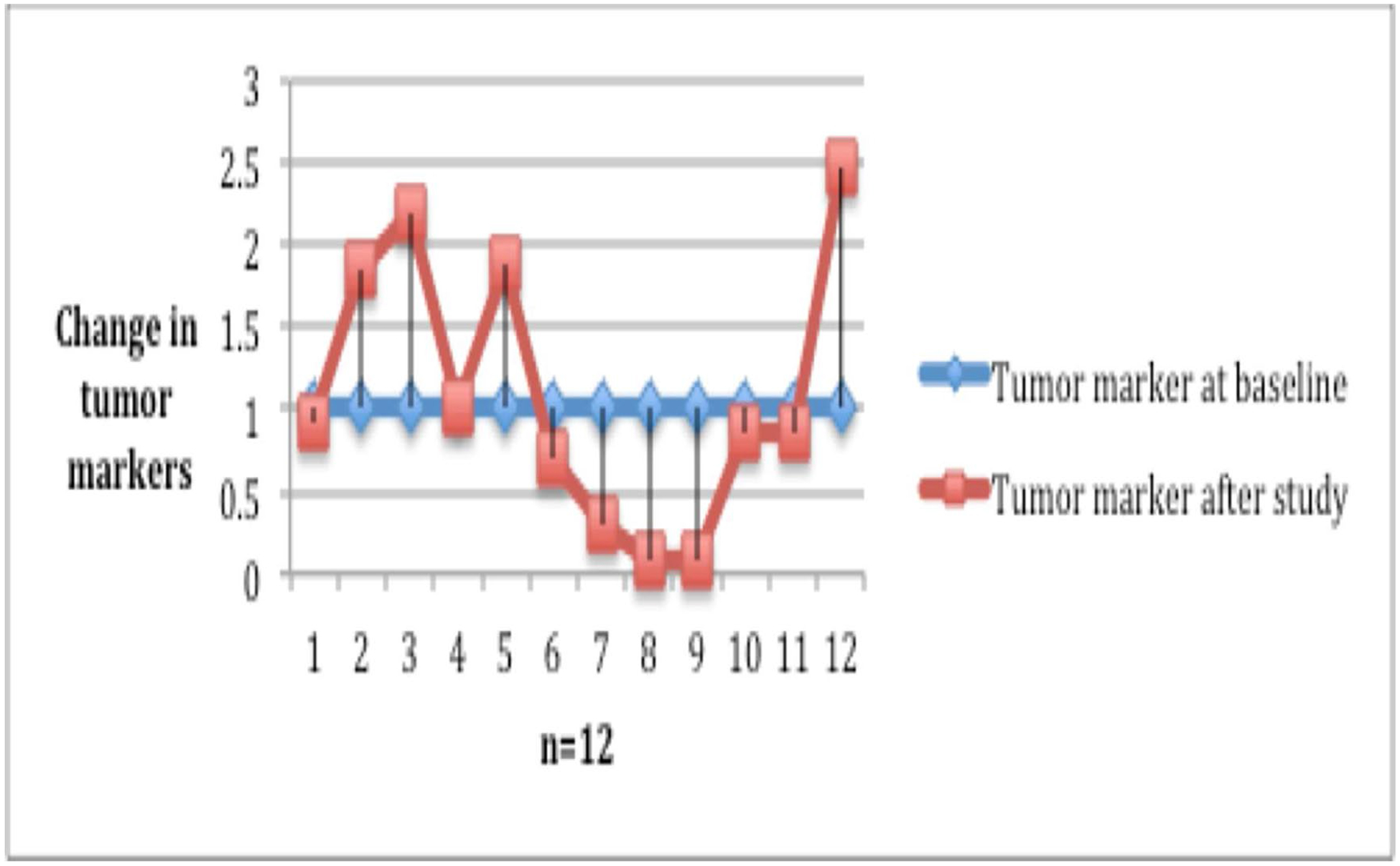
Response in tumor markers. Tumor markers (CA-19–9, CEA) were measured at baseline and subsequent treatment visits after completion of treatment. Baseline and follow-up tumor markers were available for 12 patients with gastrointestinal malignancies.

Objective treatment responses were seen across all GI malignancies, especially patients with advanced pancreatic carcinoma. Evaluation of response by imaging showed stable disease in 19 (55%) of the patients at cessation of metformin (Figure 2). Eight of the 12 patients with advanced pancreatic cancer showed disease control at the completion of study. Forty-five percent of the patients with measurable tumor markers showed improvement (Figure 3).

## Discussion

This sub-group of patients with GI cancers from our phase I study of 41 non-diabetic patients with different GI cancers showed that the rate of DLTs in patients receiving metformin in addition to chemotherapy was not higher than the DLTs in patients receiving chemotherapy alone (5.2% vs. 22.7%) in Stage 1. Our sub-group constitutes the first human data that prospectively demonstrates the impact of metformin on AMPK phosphorylation. Translational correlates included post-metformin increase in AMPK phosphorylation that may potentially explain lack of disease progression in nearly half of our patients.

Metformin has years of human experience to treat type 2 diabetes mellitus and polycystic ovary syndrome. Recently, epidemiological studies and meta-analyses have revealed that diabetic patients on metformin had a lower incidence of cancers versus than healthy controls [1–4]. Moreover, patients on metformin and diagnosed with cancer have a lower risk of mortality, further demonstrating an association between metformin and tumorigenesis.

In vivo and in vitro studies have revealed that metformin has a direct antitumor effect. Many mechanisms have been suggested to establish the antitumor effect of metformin, including reducing insulin and insulin-like growth factor levels in the peripheral blood circulation may lead to the inhibition of phosphoinositide 3-kinase/Akt/mechanistic target of rapamycin (mTOR) signaling, or activation of AMP-activated protein kinase, which inhibits mTOR signaling [5,6]. It is believed that metformin modulates AMPK, which enhances cancer stem cell killing and delay/prevent tumor xenograft re-growth when combined with a chemotherapeutic agent [11–13]. Metformin has been shown to have anti-proliferative activity against colorectal cancer cell lines, and this effect is most prominent in the p53−/−setting. Metformin suppresses polyp growth in ApcMin/+ Mice, and an important link between AMPK/glucose metabolism and colorectal cancer is the observation that Peutz-Jeghers syndrome involves a mutation in the LKB1 tumor suppressor gene, and the finding that LKB1 acts through AMPK for signaling [14]. This sub-group analysis on patients with GI cancers revealed an increase in AMPK phosphorylation on metformin, further reassuring the potential role of metformin in preventive and treatment settings in GI malignancies. Our study showed the feasibility of adding metformin to chemotherapy and another researcher showed that addition of metformin may even allow us to administer a lower dose of chemotherapy when combined with metformin, leading to less toxicities of chemotherapy [7,15]. We suggest that metformin warrants further investigation in adequately powered prospective studies. Hypoglycemia and lactic acidosis were not seen in these patients when treated with metformin, offering an additional benefit [16].

## Conclusions

In summary, this phase I study of 41 non-diabetic patients with different GI cancers showed that the rate of DLTs in patients receiving metformin in addition to chemotherapy was not higher than the DLTs in patients receiving chemotherapy alone (5.2% vs. 22.7%) in Stage 1. Our study is the first human study that prospectively demonstrates the impact of metformin on AMPK phosphorylation and revealed an increase in AMPK phosphorylation on metformin, further reassuring the potential role of metformin in preventive and treatment settings in GI malignancies. Potential benefits of metformin in preventive and treatment settings warrant further investigation in adequately powered prospective studies. This study suggests that metformin can be given safely with chemotherapy in patients with GI malignancies and offers a platform for future studies.

## Figures and Tables

**Table 1:** Baseline characteristics and treatment.

	Concurrent (N=19)	Delayed (N=22)
**Gender**		
Male	11 (57%)	13 (59%)
Female	8 (43%)	9 (41%)
**Primary Cancer**		
Colorectal	12	10
Gastroesopageal	2	2
Pancreas	4	6
Unknown Adenocarcinoma	1	1
Cholangiocarcinoma	0	2
Other	0	1
**Chemotherapy agents**		
Oxaliplatin	4	2
Capecitabine	9	11
5-FU	5	4
Bevacizumab	5	6
Carboplatin	0	3
Gemcitabine	3	7
Erlotinib	0	1
Irinotecan	5	4
Paclitaxel	3	2

**Table 2:** Dose Limiting Toxicities (DLT) during Stage 1 and Stage 2.

	Run-in stage	Stage 1	Stage 2
	Chemo (C) without DLT	Chemo + metformin (C + M) vs. Chemo (C)	Chemo + metformin (C + M)
**Concurrent**		N= 19	
	N= 19		N= 16
	None	1=G_3_ anemia, ↓ albumin, (5–2%)	N/A
**Delayed**	N= 22	N= 22	N= 21
	1=G_3_ Fatigue	1=G_3_ Anemia,	1=G_3_ dehydration, vomiting
		1=G_4_ Thrombocytopenia	
		1=G_3_ Hypoalbuminemia	
		2=G_3_ elevation of AST/ALT/bili	
	Overall: 4.5%	Overall: 22.7%	Overall: 4.8%

N: Number; G: Grade

**Table 3:** Adverse Toxicities, at least Grade 3 or higher.

	Run-in stage	Stage 1	Stage 2
	Chemo (C) without DLT	Chemo + metformin (C + M) vs. Chemo (C)	Chemo + metformin (C + M)
**Concurrent**	N= 19	N= 19	N= 16
		1=G_3_ PE, DVT	1=G_3_ hyokalemia
		1=G_3_ Infection	
		1= G_3_ anemia, hypoalbuminemia	
		Overall: 15.8%	Overall: 6.2%
**Delayed**	N= 22	N=22	N=21
		1=G_4_ HUS	1=G_3_ Surgical Hernia repair
		2=G_3_ AST/ALT/bili	1=G_3_ Dehydration
		Overall: 13.6%	Overall: 9.5%

N= number, G=grade
